# Effectiveness of brief online mindfulness-based intervention on different types of mobile phone addiction: mechanisms of influence of trait mindfulness

**DOI:** 10.3389/fpsyg.2025.1400327

**Published:** 2025-02-17

**Authors:** Zhiyu Zhang, Lei Wu, Chu Lu, Tianming Guan

**Affiliations:** ^1^Faculty of Psychology, Tianjin Normal University, Tianjin, China; ^2^Key Research Base of Humanities and Social Sciences of the Ministry of Education, Academy of Psychology and Behavior, Tianjin Normal University, Tianjin, China

**Keywords:** mindfulness, brief online mindfulness-based intervention, mobile phone addiction, trait mindfulness, hierarchical linear modeling

## Abstract

**Introduction:**

It is common for college students to suffer from mobile phone addiction, which can seriously affect their physical and mental health. The current study looked at the impact of a brief online mindfulness-based intervention (MBI) on mobile phone addiction to address this problem. The mechanisms behind the influence of trait mindfulness (TM) on this process were also investigated in the current study.

**Methods:**

Subjects were split into two groups for a randomized controlled trial: an experimental group and a control group. During brief online MBI, online assessments of mobile social networking addiction (MSNA), mobile game addiction (MGA), mobile information acquisition addiction (MIAA), and mobile short-form video addiction (MSVA) as well as TM were completed. The present study examined the effectiveness of the brief online MBI with a two-factor repeated measures ANOVA and explored the effects of TM on the four types of mobile phone addiction with hierarchical linear modeling (HLM) after a brief online MBI.

**Results:**

Results revealed that the experimental group receiving brief online MBI showed a significant decrease in MSNA, MGA, MIAA, and MSVA at the post-test level compared to the pre-test. TM negatively predicted MSNA, MGA, MIAA, and MSVA.

**Discussion:**

The present study found that the brief online mindfulness-based intervention can effectively reduce four types of mobile phone addiction. In addition, the present study revealed that as the levels of TM increased, all four types of mobile phone addiction decreased.

## Introduction

Everybody’s life now revolves around their mobile phone, however many are struggling with mobile phone addiction. According to Liu et al. (2022b) there are major physiological, psychological, and social deficits associated with mobile phone addiction, which is characterized as a combined condition of excessive psychological need and usage of mobile phones. Due to their immaturity and lack of self-control, college students are more prone than members of older social groups to suffer from mobile phone addiction (Li et al., 2018; Long et al., 2016). A meta-analysis found that over 23% of Chinese college students suffer from a mobile phone addiction on average (Tao et al., 2018). Numerous college students are addicted to their phones, which has numerous negative consequences. In three-year longitudinal research conducted in China using college students as subjects, Zhang et al. (2020) discovered that poor mental health status (i.e., anxiety, depression, and subjective well-being) at Year 3 was strongly predicted by mobile phone addiction at Year 1. A meta-analysis of Korean college students revealed that behavioral procrastination and impulsivity, a decline in social connections, loneliness, and suicidal thoughts were among the detrimental consequences of mobile phone addiction on college students (Achangwa et al., 2023). College students’ physical and mental health have been negatively impacted by the frequency and intensity of mobile phone addiction. Finding treatments that are effective for college students who are addicted to their phones has therefore gained importance.

In the past few decades, mindfulness has been developed as a mental training skill and utilized in numerous interventions for treating behavioral and substance addictions. It has been proven to be highly effective in a significant number of studies (Garland and Howard, 2018; Rosenthal et al., 2021; Sancho et al., 2018). Traditional mindfulness-based interventions (MBI) include eight two-hour weekly in-person group sessions, a full day of practice, meditation logs, and journaling tasks as homework (Kabat-Zinn, 2003). Certain experts have proposed that dosage is a critical factor to consider when choosing a treatment plan because varying amounts can have distinct effects. To save treatment costs and time, the mini-effective dose is recommended to use (Garland and Howard, 2018). Confounding variables resulting from long-term MBIs may exist (Liu et al., 2022a). In addition, due to the continuous development of technology, the accelerating pace of life, and the global outbreak of COVID-19, the global population is facing long-term home isolation and cannot engage in offline activities. In this context, researchers are exploring the possibility of brief online MBIs as an alternative. Brief online MBI is defined as an intervention that lasts no more than 30 min on any given day, no more than 100 min weekly, and up to 4 weeks (Jiménez et al., 2020). It involves self-guidance, flexibility, and anonymity, and has been proven effective in numerous studies. It also shortens the intervention period and overcomes spatial constraints (Cavanagh et al., 2018; Villalón et al., 2023; Zhang et al., 2021). However, few studies have confirmed the usefulness of brief online MBI on mobile phone addiction, despite the fact that MBI has been shown to lower the level of addiction to mobile phones (Lan et al., 2018; Liu et al., 2022a). Thus, the current study will look at how short-term online mental health interventions affect addiction to mobile phones.

Trait mindfulness (TM) refers to an individual’s general tendency to remain aware of and present in the moment, characterized by a non-judgmental and accepting attitude toward one’s thoughts, emotions, and sensations. TM is a stable, enduring trait that reflects a person’s habitual capacity to engage in mindful awareness across various situations (Brown and Ryan, 2003). Individuals high in TM tend to approach experiences with greater attentiveness, emotional regulation, and cognitive flexibility, enabling them to respond to challenges, including addictive behaviors, with greater awareness and control. Higher levels of TM help individuals reduce impulsive behaviors, as they are better at recognizing and managing their automatic reactions (Kang et al., 2013). This ability to regulate impulses is particularly relevant in the context of mobile phone addiction, as such addiction is often driven by immediate gratification from phone use. Individuals with higher TM are less likely to engage in these automatic, compulsive behaviors and are more capable of making intentional decisions about their phone usage (Woodlief, 2017). Research has shown that mobile phone addiction levels have been demonstrated to be negatively predicted by TM (Jin et al., 2023; Yang et al., 2023). The relationship between TM and mobile phone addiction is also relevant in the context of brief online MBIs. While brief online MBI have been shown to reduce mobile phone addiction (Hefner and Freytag, 2024). In longitudinal studies, the potential role of TM in this process has not been fully explored. This study aims to close the gap in the literature by examining the roles of TM in mobile phone addiction during a brief online MBI.

Numerous researchers have raised concerns, conducted studies, and drawn the conclusion which had shown that mobile phone addiction should not be regarded as an independent substance addiction, but rather as a facilitator for other behavioral addictions. With the mobile phone, it is possible to access different types of apps and websites, which can develop into different types of mobile phone addictions (Carbonell et al., 2022; Davazdahemami et al., 2016), and Marino emphasizes that in studies of mobile phone use, a distinction should first be made between different types of apps rather than examining mobile phone addiction as a whole (Marino et al., 2021). Nonetheless, the majority of recent research has focused on mobile phone addiction per se, with very few studies having investigated the many forms of mobile phone addiction. Liu developed the Mobile Phone Addiction Types Scale (MPATS) to categorize addiction to mobile phone use into four different types after interviewing 108 Chinese university students and testing the reliability of the MPATS on 854 adolescents in 2022, namely mobile social networking addiction (MSNA), mobile game addiction (MGA), mobile information acquisition addiction (MIAA), and mobile short-form video addiction (MSVA) (Liu et al., 2022a,b). Therefore, the present study will examine the four Mobile Phone Addiction Types Scale using the MPATS questionnaire with the four mobile phone addictions as targets. Although Liu’s follow-up questionnaire using the MPATS questionnaire with 1,202 adolescents found that TM negatively predicted MSNA, MGA, MIAA, and MSVA (Liu et al., 2022b), the study used a cross-sectional study to draw its conclusions. It did not conduct a longitudinal follow-up study, and second, as mentioned in the previous paragraph, the study did not examine the relationship between state mindfulness and the four types of mobile phone addiction. Moreover, Hefner and Freytag (2024) discovered that individuals’ habitual mobile phone use was successfully decreased by both an eight-week traditional in-person mindfulness training and a three-week online mindfulness training, with sessions lasting 7–10 min each day. Nevertheless, no research has yet looked at how TM affects various forms of mobile phone addiction in the framework of a brief online MBI. This work aims to solve this problem.

In summary, this study aims to examine the effectiveness of a brief online MBI on MSNA, MGA, MIAA, and MSVA, and to investigate the mechanisms by which TM influence these four types of mobile phone addiction in this process. The hypotheses of this study are: (1) the brief online MBI will reduce the levels of MSNA, MGA, MIAA, and MSVA, and (2) TM will negatively predict the four types of mobile phone addiction during the brief online MBI.

## Materials and methods

### Subjects

The present study began on April 25, 2023, with the release of a teaser poster titled ‘Mindfulness-Based Intervention’ on WeChat Moments, QQ Zone, and online campus platforms. Subjects interested scanned the QR code questionnaire, completed demographic and baseline measures, and were screened for eligibility. To ascertain eligibility, interested participants were sent to an online screening questionnaire. Subjects had to reach the threshold score for mobile phone addiction (>31 for men and > 33 for women) on the Chinese version of the Smartphone Addiction Scale in order to be eligible for inclusion (Kwon et al., 2013; Zhao et al., 2022). The absence of a serious physical illness, cognitive disability, or prior experience with mindfulness or meditation were the exclusion criteria. Participants in the current study were invited if they satisfied the inclusionary and exclusion criteria. Written informed permission was obtained from participants before to the start of the current study, and their participation would be kept personal and anonymous. This study was authorized by Tianjin Normal University’s Ethical Review. All subjects were informed about the study and all provided informed consent.

A prior power analysis using G*Power 3.1 with parameters set at an effect size of 0.25, alpha 0.05, and power 0.95, yielded a total sample size of 54. 104 college students (average age = 20.82, SD = 1.99; 21 males and 83 females) participated in the current study. As a result, a sufficient sample size was chosen. The participants were randomly allocated to either the experimental group (n = 59; 13 males and 46 females) or the control group (n = 45; 10 males and 35 females), taking into consideration the high task volume of the experimental group and the potentially high rate of subject dropout. The total number of subjects who dropped out during the experiment or participated in less than 50% of the activities was 3. The final remaining experimental group was 57 (12 males and 45 females) and the control group 44 (*n* = 45; 10 males and 34 females).

### Procedure

In this investigation, a randomized controlled trial (RCT) was employed. Using a random number table, each participant was randomized to either the experimental group, which received the intervention, or the control group, which did not. We introduced the experimental and control groups to the relevant WeChat group on May 7 and went over the definition of mindfulness, the risks associated with mobile phone addiction, and the brief online MBI plan. Before baseline data were collected, online informed permission was acquired from every participant. Subjects in the control group did not receive any intervention during the experimental group’s intervention and were instructed to begin the intervention after 30 days. In contrast, subjects in the experimental group were asked to participate in live MBI via the “Tencent Meeting” software at 9:00 p.m. every day for 30 consecutive days. To ensure that participants who missed the live session could still complete the training on the same day, we sent the recorded live stream to the WeChat group. All participants in the experimental group were able to follow the recorded session at any time on the same day to complete the mindfulness training. All data were automatically gathered over the Internet. At the end of every 3 days of intervention, they completed a measure of the four types of mobile phone addiction and TM, whereas subjects in the control group received a link to the questionnaire in the WeChat group at the same time and completed the same measures. The questionnaires were counted as invalid if they were sent back over 24:00 on the day. After the intervention was completed, we conducted a collection and organizing process for the questionnaires. A total of 11 measurements were conducted including the baseline measurement.

### The brief online MBI

The Wherever You Go, There You Are: Mindfulness Meditation in Everyday Life book that Kabat-Zinn authored (Kabat-Zinn, 1994) and the Positive Mindfulness-Based for Stress Reduction (MBSR) program that he developed in 2003 are the main sources of inspiration for the brief online MBI created for this study. The original MBSR program consisted of weekly 2.5-h group sessions for 8 weeks plus a half-day retreat. On the other hand, in order to confirm the significant effects of brief online MBI, we purposefully decreased the intensity and duration of the original MBSR in the current study.

In the present study, subjects were asked to participate in a live online session through the “Tencent Meeting” software and to follow the instructions of a mindfulness leader for about 10 min each time. The leader was a graduate student in applied psychology, and the guidelines were reviewed by a professor with rich experience in MBIs and edited based on her feedback, resulting in a guideline text size of about 280 Chinese characters. Breath-counting, breath-watching, and body scanning are considered to be the first stage of MBIs and are more suitable for beginners (Liu et al., 2013; Rowland et al., 2019; Somaraju et al., 2023). Therefore, the three forms described above were chosen as the brief online MBI to be practiced in the present study. Breath-counting on the first 9 days, breath-watching on days 10 through 19, and body scanning on days 20 through 30.

Breath Counting, that breath-out and breath-in are counted from 1 to 10, focuses on the sensations associated with breathing. Then, if you have a vague mental image of the number or are aware of yourself thinking about something else, start counting from one again. The Breath Observation focuses on feeling the changes in your nasal passages or abdomen as you breathe and being aware of the content of your thoughts at all times. Body Scanning focuses on the main fatigue areas of mobile phone addicts (waist, back, shoulders, neck, and eyes) and combines breathing with awareness and relaxation intervention.

### Measures

#### Mobile phone addiction

The Chinese version of the Smartphone Addiction Scale-Short Version (Kwon et al., 2013; Zhao et al., 2022) was employed for measurement. The scale contains a total of 10 questions. An example question is “Won’t be able to stand not having a smartphone.” The Likert 6-point rating system, which ranges from 1 to 6 to indicate degrees of variance from Strongly Disagree to Strongly Agree, is used for all titles. Higher scores correspond to more addiction to mobile phones. The questionnaire’s internal consistency reliability in the current investigation is 0.87.

#### Four types of mobile phone addiction

The Chinese version of the Mobile Phone Type Addiction Scale (Liu et al., 2022b) was employed for measurement, it consists of four subscales that measure MSNA, MGA, MIAA, and MSVA. The scale contains a total of 26 questions, including 6 questions in the MSNA, 6 questions in the MGA, 7 questions in the MIAA, and 7 questions in the MSVA. Sample questions include “I cannot stand not looking at the social apps on my phone for a while,” “I think the amount of time I spend playing mobile games each day is too short,” “Even though the information is irrelevant, I still have a hard time controlling myself from searching and browsing on my phone,” and “It is hard for me to last long without viewing short-form videos on my phone, even if it is just for a few hours.” The questions were graded using a 5-point Likert scale, where “never” to “always” was represented by a score of “1” through “5.” The internal consistency reliability of the questionnaire in this study varied from 0.94 to 0.98.

#### Trait mindfulness

The Chinese version of the Mindful Attention Awareness Scale (MAAS) (Brown and Ryan, 2003; Deng et al., 2012) was employed for measurement. The scale contains a total of 10 questions. An example question is “I may be experiencing some emotions without being conscious of them until sometime later.” The titles all adopt the Likert 6-point scoring method, where 1 to 6 represents degrees of variation from “Almost Always” to “Almost Never.” Higher TM are indicated by higher scores. The internal consistency reliability of the questionnaire in this study varied from 0.85 to 0.94.

### Data analyses

**Figure 1:**
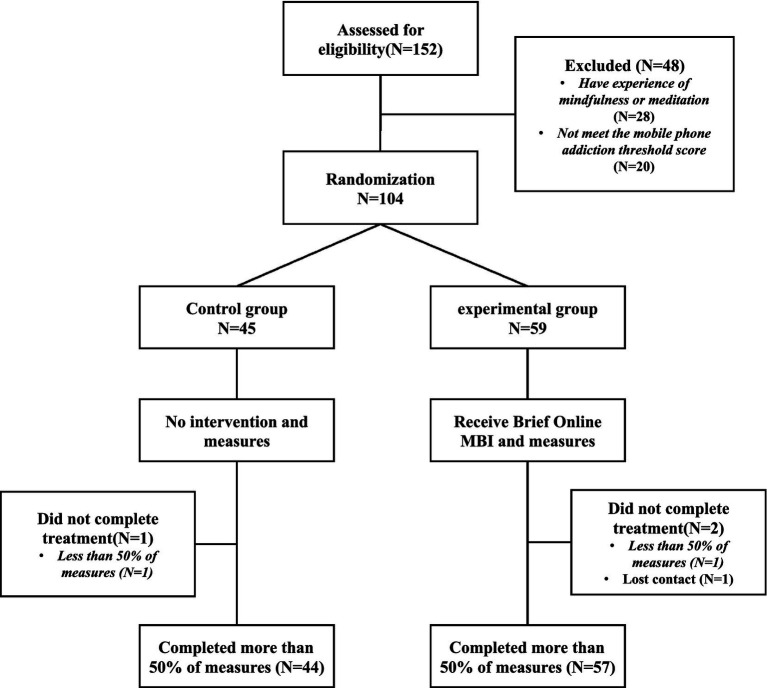
CONSORT flow diagram.

Figure 1 shows the study flow. The present study began with the variables measured at baseline, including MSNA, MGA, MIAA, MSVA, and TM. Independent *t*-tests were conducted on the subjects to assess the differences between the two groups before the experiment.

Following the intervention, a two-factor repeated measures ANOVA (group: experimental group vs. control group) × 2 (time: pre-test vs. post-test) was conducted separately for the four variables (MSNA, MGA, MIAA, and MSVA) and TM to evaluate the differences between the two groups before and after the intervention during the assessment points. Significant interactions were followed by Bonferroni *post hoc* tests. We used an intention-to-treat analysis, in which all subjects randomly assigned to both groups were statistically analyzed, and chose multiple interpolations for posttest missing values, which is currently considered a more accurate method for dealing with missing values in repeated measures data (McCoy, 2017).

Finally, we used hierarchical linear modeling (HLM) and created a full model that included measurement time, group (experimental group vs. control group), TM, and interactions between time and group. HLM analyzed data from all 11 measurements. In the HLM, the within-individual variable is placed in the first-level regression equation and the between-individual variable is placed in the second-level regression equation. To create the first-level regression equation in the HLM analysis, we first set the four forms of mobile phone addiction as the dependent variables and measurement time (categorical variable) as the independent variable. We then created the second-level regression equation utilizing group (categorical variable) and TM as independent variables and the intercept and slope of the first-level model as dependent variables. Therefore, we constructed four HLMs, with the dependent variables of these HLMs being MSNA, MGA, MIAA, and MSVA, respectively.

Compared to more traditional methods (such as analysis of variance), HLM was performed separately for each of the assessed indexes and simultaneously considering the effectiveness of within-individual and between-individual variables on the dependent variables. Measurement data from different time points can all be included in the analysis. This approach provides more accurate results regarding the intervention effects and allows for handling non-homogeneity of variance and missing data (Hox et al., 2017).

## Results

### Baseline

We first conducted an independent samples *t*-test on the scores of the pre-test questionnaires administered to the experimental group and control group to examine whether there was any significant difference between the two groups before the intervention. As shown in Table 1, there was no significant difference between the experimental and control groups in the pre-test mobile phone addiction, MSNA, MGA, MIAA, MSVA, and TM (*p* > 0.05). The result of the present study suggests that the two groups are homogeneous to some extent and both fulfill the criteria for mobile phone addiction.

**Table 1:** Descriptive statistics and *t*-test for all variables at baseline.

	EG		CG		*F*	*p*	*t*
	*M*	*SD*	*M*	*SD*			
MPA	43.26	8.31	43.75	6.60	1.47	0.751	−0.32
MSNA	21.70	4.11	21.46	4.31	0.04	0.770	0.29
MGA	14.56	4.79	15.32	4.64	0.37	0.427	−0.80
MIAA	24.98	5.81	25.93	4.49	3.12	0.372	−0.90
MSVA	24.19	5.72	23.59	6.45	0.35	0.621	0.50
TM	53.23	11.71	55.27	9.88	0.73	0.354	−0.93

MPA, mobile phone addiction; MSNA, mobile social networking addiction; MGA, mobile phone game addiction; MIAA, mobile information acquisition addiction; MSVA, mobile short-form video addiction; EG, experimental group; CG, control group.

### The effectiveness of brief online MBI

To test the effectiveness of the brief online MBI, 2 (group: experimental group vs. control group) × 2 (time: pre-test vs. post-test) two-factor repeated measures analysis of variance was conducted with MSNA, MGA, MIAA, MSVA, and TM.

**Figure 2:**
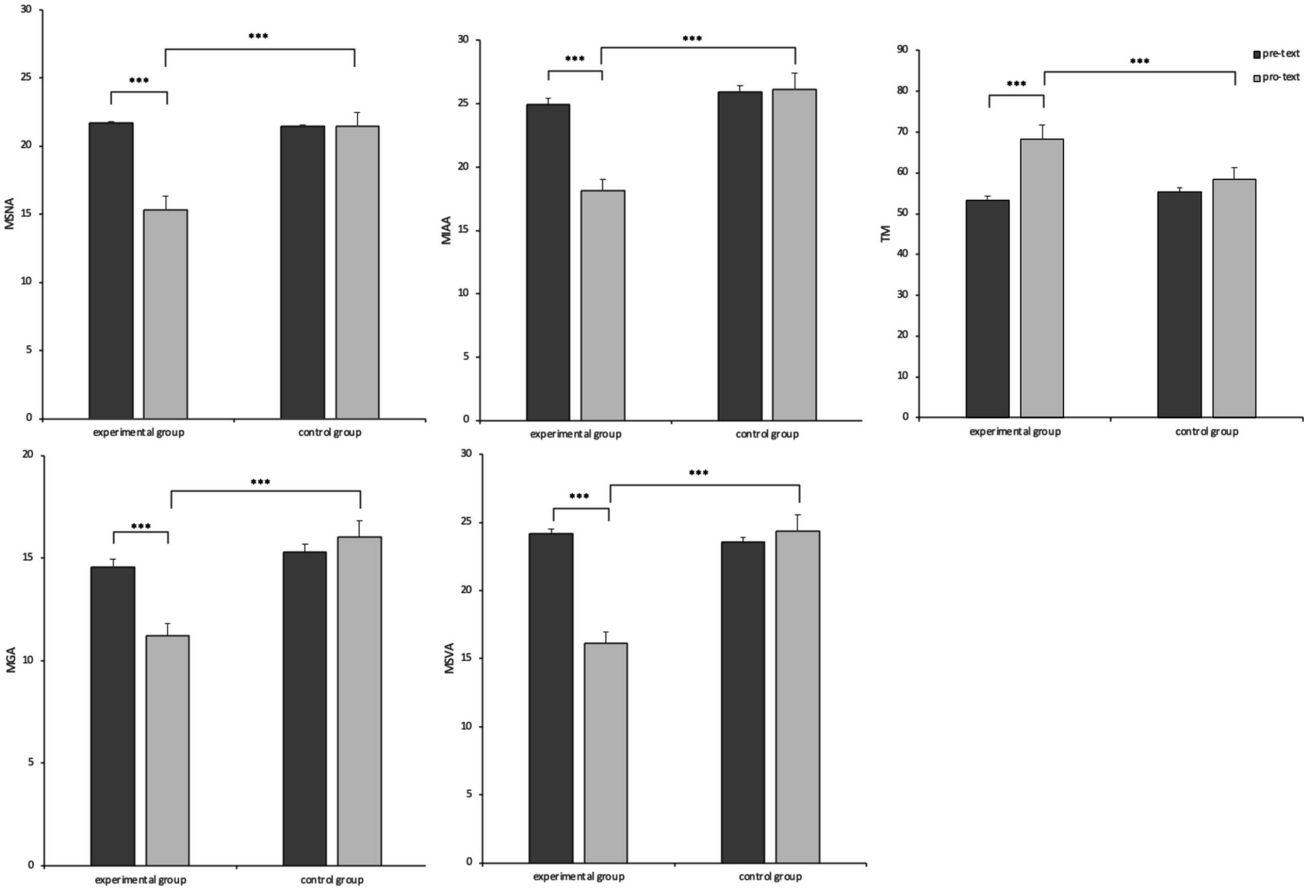
The four types of mobile phone addiction and TM between pre- and post-text. ***p > 0.001.

The results, as shown in Table 2, showed significant main effects of group and time of measurement for all dependent variables, as well as significant interactions of group and time of measurement. Further simple effects analyses were conducted, as shown in Figure 2, the experimental group had MSNA, *F*(1, 99) = 117.62, *p* < 0.001, $\eta_{p}^{2}$ = 0.543, MGA, F(1, 99) = 20.08, *p* < 0.001, $\eta_{p}^{2}$ = 0.169, MIAA, F(1, 99) = 65.69, *p* < 0.001, $\eta_{p}^{2}$ = 0.399 and MSVA, F(1, 99) = 114.64, *p* < 0.001, $\eta_{p}^{2}$ = 0.537 were significantly lower at the posttest level than at the pre-test; the experimental group had TM, F(1, 99) = 3.68, *p* < 0.001, $\eta_{p}^{2}$ = 1.36 were significantly higher at the posttest level than at the pre-test. In contrast, all dependent variables in the control group were not significantly different at the pre-test and post-test levels. The above results indicate that brief online MBI affects MSNA, MGA, MIAA, MSVA, and TM. The *post hoc* tests are shown in Figure 2.

**Table 2:** Descriptive statistics for four types of mobile phone addiction and TM post-intervention.

	EG		CG		Time		$\eta_{p}^{2}$	Group		$\eta_{p}^{2}$	T × G		$\eta_{p}^{2}$
	*M*	*SD*	*M*	*SD*	*F*	*p*		*F*	*p*		*F*	*p*	
MSNA	15.32	3.68	21.46	4.42	70.00	0.000	0.414	16.22	0.000	0.141	70.26	0.000	0.415
MGA	11.24	3.79	16.04	5.86	6.91	0.010	0.065	11.52	0.001	0.104	16.73	0.000	0.145
MIAA	18.12	4.86	26.11	4.46	33.39	0.000	0.252	29.82	0.000	0.231	36.95	0.000	0.272
MSVA	16.15	4.85	26.35	6.17	45.11	0.000	0.313	13.92	0.000	0.123	65.89	0.000	0.400
TM	68.33	10.51	58.34	12.30	114.60	0.000	0.537	3.68	0.050	0.036	50.08	0.000	0.336

MSNA, mobile social networking addiction; MGA, mobile phone game addiction; MIAA, mobile information acquisition addiction; MSVA, mobile short-form video addiction; TM, trait mindfulness; EG, experimental group; CG, control group. T × G, Time × Group interaction.

### Mechanisms of the effects of TM on four types of mobile phone addiction

In the initial phase, the intraclass correlation coefficient (ICC) was computed to evaluate the lack of independence of observation. The null model (intercept-only model) was used to do this, and the between-cluster variance was divided by the total of the within- and between-cluster variances. If ICC is greater than 0.25, it indicates that the dependent variable exhibits both between-individual and within-individual variations, making it suitable for HLM (Cohen et al., 2003). The results reveal that the ICC for MSNA, MGA, MIAA, and MSVA are 0.79, 0.72, 0.66, and 0.75, respectively. The result of the present study suggests that these variables are suitable for HLM.


$$\begin{array}{l} \mathrm{LEVEL}\;1\;\mathrm{MODEL}\\ Y_{ij}=\beta_{0j}+r_{ij}\\ \mathrm{LEVEL}\;2\;\mathrm{MODEL}\\ \beta_{0j}=\gamma_{00}+\mu_{0j} \end{array}$$

Secondly, we constructed the full model with MSNA, MGA, MIAA, and MSVA as dependent variables by taking the within-individual variable including measurement time points (coded 0 to 10) as the first level of predictor variables, and the between-individual variable including group (coded 0 and 1) and TM as the second level of predictor variables. γ_00_ represents the grand mean of dependent variables. R and U_0_ represent the residual terms of the first and second levels of the model, respectively.


$$\begin{array}{l} \begin{array}{l} \mathrm{LEVEL}\;1\;\mathrm{MODEL}\\ Y_{ij}=\beta_{0j}+\beta_{1j}\left(\mathrm{TIME}-{\bar{\mathrm{TIME}.}}_{j}\right)+r_{ij}\\ \mathrm{LEVEL}\;2\;\mathrm{MODEL}\\ \beta_{0j}=\gamma_{00}+\gamma_{01}\left(\mathrm{GROU}P_{j}\right)+\gamma_{02}\left(TM_{j}\right)+\mu_{0j} \end{array}\\ \beta_{1j}=\gamma_{10}+\gamma_{11}\left(\mathrm{GROU}P_{j}-\bar{\mathrm{GROUP}.}\right)+\gamma_{12}\left(TM_{j}-\bar{TM.}\right)+\mu_{1j} \end{array}$$

Detailed results are presented in Table 3, measurement time had a significant negative predictive effect on MSNA (ß = −0.117, *p* < 0.001), MGA (ß = −0.074, *p* < 0.001), MIAA (ß = −0.117, *p* < 0.001), and MSVA (ß = −0.131, *p* < 0.001). Additionally, there was a significant difference between the EG and the CG on MSNA (ß = −3.224, *p* < 0.001), MGA (ß = −3.326, *p* < 0.001), MIAA (ß = −4.045, *p* < 0.001), and MSVA (ß = −4.283, *p* < 0.001). Four types of mobile phone addiction of CG were significantly higher than that of EG. An interaction between measurement time and group negatively predicted MSNA (ß = −0.181, *p* < 0.001), MGA (ß = −0.144, *p* < 0.001), MIAA (ß = −0.227, *p* < 0.001), and MSVA (ß = −0.225, *p* < 0.001). This result was identical to the conclusion reached by the repeated measures ANOVA with pre-test and post-test as independent variables. More importantly, TM had a significant negative predictive effect on MSNA (ß = −0.140, *p* < 0.001), MGA (ß = −0.104, *p* < 0.05 = 0.015), MIAA (ß = −0.225, *p* < 0.001), and MSVA (ß = −0.149, *p* < 0.01 = 0.007).

**Table 3:** Hierarchical linear model for four types of mobile phone addiction.

	MSNA		MGA		MIAA		MSVA	
	Null model	Full model	Null model	Full model	Null model	Full model	Null model	Full model
Fixed effect								
Level 1								
Intercept (γ_00_)	19.465***	19.461***	13.603***	13.599***	23.014***	23.009***	21.128***	21.125***
Time (γ_10_)		−0.117***		−0.074***		−0.117***		−0.131***
Level 2								
Group (γ_01_)		−3.224***		−3.326***		−4.045***		−4.283***
TM (γ_02_)		−0.140***		−0.104*		−0.225***		−0.149**
Interaction effect								
Time × Group (γ_11_)		−0.181***		−0.144***		−0.227***		−0.225***
Random effect								
U_0_	15.273***	10.500***	4.429***	15.806***	4.829***	13.345***	5.568***	24.111***
R	7.77	4.93	2.78	5.19	3.48	7.66	3.2	6.00
Deviance	5215.72	4881.86	5234.26	5016.53	5659.11	5330.03	5533.14	5165.29

MSNA, mobile social networking addiction; MGA, mobile phone game addiction; MIAA, mobile information acquisition addiction; MSVA, mobile short-form video addiction; TM, trait mindfulness.

*p < 0.05, **p < 0.01, ***p < 0.001.

## Discussion

Mobile phone addiction is a prevalent problem among college students that can lead to serious risks and consequences. Therefore, it is important to find effective ways to intervene. This study demonstrates the effectiveness of a brief online MBI in reducing different types of mobile phone addiction among college students. To enhance comprehension of the many forms of mobile phone addiction, we have classified it into four categories: MSNA, MGA, MIAA, and MSVA (Carbonell et al., 2022; Lowe-Calverley and Pontes, 2020). To assess the impact of brief online MBI on these four categories of mobile phone addiction, we carried out a randomized controlled experiment. We also looked at how TM predicted the four different forms of mobile phone addiction. Overall, the brief online MBI is effective in reducing mobile phone addiction and changes in TM. Additionally, TM can help decrease the level of mobile phone addiction. The results of this study offer a solid scientific foundation for the treatment and prevention of mobile phone addiction.

First of all, results from the repeated measures ANOVA indicated that, compared to the pre-test, the experimental group that received the brief online MBI exhibited significant decreases in the levels of MSNA, MGA, MIAA, and MSVA during the post-test, whereas the untrained control group showed no changes in these levels. These findings suggest that a brief online MBI, conducted for 10 min per day over 30 days, is effective in reducing the four types of mobile phone addiction behaviors. Additionally, based on the results of HLM analysis of data measured every 3 days, this study found that the interaction between time and group significantly negatively predicted MSNA, MGA, MIAA, and MSVA. As a result, in the experimental group, the levels of all four types of mobile phone addiction were significantly lower at each subsequent time point compared to the control group. In other words, during the course of the brief online MBI, the levels of the four types of mobile phone addiction in the experimental group, which underwent the intervention, were significantly lower than those in the control group every 3 days. Compared to the results obtained from two-factor repeated measures analysis of variance, HLM provides the advantage of monitoring changes every 3 days throughout the intervention, rather than only comparing the baseline and the 30-day time point. More importantly, HLM allows for the simultaneous examination of the effects of both between-individual and within-individual variables on the dependent variable. In contrast, two-factor repeated measures ANOVA can only evaluate the effect of one independent variable on the dependent variable by fixing the other through simple effects analysis (Hox et al., 2017). In this study, HLM enabled the observation of the combined effects of time and group on mobile phone addiction. However, two-factor repeated measures ANOVA could only separately analyze the differences in mobile phone addiction levels between the pre-test and post-test within the experimental group and the control group, without allowing for a direct comparison of group differences simultaneously. Previous studies provide robust theoretical support for the current findings. For example, Lan et al. (2018) demonstrated that traditional MBI effectively reduce individuals’ levels of mobile phone addiction. Similarly, Wang and Chen (2023) conducted an eight-week mindfulness therapy intervention among Chinese adolescents and found that the experimental group exhibited significantly lower levels of mobile phone addiction compared to the control group. These findings are consistent with our results, our research further shows that a brief online MBI is beneficial for all four forms of mobile phone addiction and can lower participants’ degrees of addiction.

This study further categorizes mobile phone addiction into four distinct types: MSNA, MGA, MIAA, and MSVA. For all four types, existing studies have examined their relationships with mindfulness. In the case of MSNA, studies indicate that mindfulness enhances individuals’ attentional control and reduces their fear of missing out, thereby reducing social media addiction (Chang et al., 2023). Regarding MGA, mindfulness strengthens self-regulation abilities, enabling individuals to better manage their gaming time and mitigate the psychological negative effects of excessive gaming (Song and Park, 2019). For MIAA, individuals’ MIAA levels tend to increase in high-social-risk environments, but mindfulness acts as a negative moderator in this relationship, lowering MIAA levels (Liu et al., 2022c). Additionally, in relation to MSVA, mindfulness allows individuals to become more aware of the emotions and impulses associated with short video use, enhancing self-control and reducing addictive behaviors (Dong et al., 2024). This explains why mindfulness is effective for all four types of mobile phone addiction. Building on these findings, our study further demonstrates that a brief online MBI is applicable to all four categories of mobile phone addiction. Moreover, this new finding provides more options for developing intervention strategies to reduce mobile phone addiction. Considering that college students are often under pressure of time constraints and heavy coursework (Hurst et al., 2012), it may be difficult for them to participate in time-consuming traditional MBI that need to be conducted offline. Therefore, brief online MBI becomes a more flexible, efficient, and less spatially restrictive solution to help address college students’ mobile phone addiction.

In addition to examining the effectiveness of the brief online MBI using mobile phone addiction as the dependent variable, we also investigated the changes in TM levels during the intervention. Results from a two-way repeated measures ANOVA revealed that, compared to the pre-test, the experimental group showed a significant increase in TM, while the control group exhibited no change in TM. This indicates that the brief online MBI is also effective in enhancing individuals’ TM levels. Previous studies have extensively demonstrated that not only traditional mindfulness training but also brief online MBIs can improve individuals’ TM levels (Jiménez et al., 2020; Verhaeghen, 2021). The brief online MBI exists in various formats, including training programs lasting from 1 week to 4 weeks, with practice sessions ranging from 7 to 15 min in duration. Notably, some studies have shown that there is no significant difference in mindfulness levels before and after a single 15-min mindfulness session (Somaraju et al., 2023). Therefore, while brief online mindfulness practices offer a good option for those with busy schedules who cannot regularly participate in longer sessions, a single short session may still be insufficient to provide meaningful changes for individuals in need of support.

Furthermore, through the results of HLM analysis, the present study investigated the mechanisms through which TM affect MSNA, MGA, MIAA, and MSVA. We found that the level of TM showed negative predictive effects on an individual’s MSNA, MGA, MIAA, and MSVA. This finding is consistent with earlier research by Liu et al. (2022b) on the relationship between TM and mobile phone addiction, as well as the conclusions drawn by Yang et al. (2023). In contrast to the cross-sectional studies above, the present study used an RCT of longitudinal intervention to validate the results. It similarly highlighted the negative predictive effects between TM and mobile phone addiction. So how does TM affect mobile phone addiction? The resource conservation theory states that people often acquire, invest in, or protect resources they value as a way to deal with stress and anxiety. In this context, “resources” refers to any qualities, things, energies, or circumstances that individuals value, including characteristic mindfulness (Öge et al., 2018). TM has been shown to promote mental clarity and attentional intensity (Hanley and Garland, 2017), lower stress and depressive mood (Jones et al., 2022), improve subjective well-being (Liu et al., 2020), and help people maintain good mental health. Psychological processes including self-compassion, emotion management, and cognitive reappraisal are how the MBI works. This bolsters the resource conservation theory’s assertion that college students who possess high TM levels are better equipped to handle complex emotions, manage risks, and focus their cognitive attention on the here and now (Keng et al., 2011). Furthermore, MBI helps people to keep an open and accepting mindset, improve their capacity to “let go” of unpleasant events, and constructively and productively concentrate on experiences—all of which effectively prevent behaviors associated with mobile phone addiction (Koo et al., 2011; Sedlmeier, 2023). This explains why elevating TM effectively diminishes the inclination toward mobile phone addiction.

### Limitations and future directions

In the present study, considering the burden on the subjects during the intervention, a frequency of measurements every 3 days was used without collecting data at a higher frequency. This may affect the precision of the results and amplify the effects of random errors. In future studies, we suggest using Ecological Momentary Assessment for assessment, such as collecting data at a frequency of once per session or three times per day, at a high density, and assessing subjects in different temporal scenarios to ensure more precise results. In addition, considering a larger sample size and examining it in conjunction with qualitative studies can make the results more generalizable and comprehensive.

## Data Availability

The raw data supporting the conclusions of this article will be made available by the authors, without undue reservation.
